# Regulation of NF-κB- and STAT1-mediated plasmacytoid dendritic cell functions by A20

**DOI:** 10.1371/journal.pone.0222697

**Published:** 2019-09-23

**Authors:** Pham Ngoc Duy, Nguyen Thu Thuy, Bui Kieu Trang, Nguyen Hoang Giang, Nguyen Thi Hong Van, Nguyen Thi Xuan

**Affiliations:** 1 Faculty of Biotechnology, Vietnam National University of Agriculture, Trau Quy, Gia Lam, Hanoi, Vietnam; 2 Institute of Biomedicine and Pharmacy, Vietnam Military Medical University, Ha Dong, Hanoi, Vietnam; 3 Institute of Genome Research, Vietnam Academy of Science and Technology, Cau Giay, Hanoi, Vietnam; 4 Department of Genetics, Faculty of Biology, VNU University of Science, Thanh Xuan, Hanoi, Vietnam; 5 Graduate University of Science and Technology, Vietnam Academy of Science and Technology, Cau Giay, Ha Noi, Vietnam; Karolinska Institutet, SWEDEN

## Abstract

Dendritic cells (DCs) are professional antigen presenting cells involved in the induction of T cell-mediated adaptive immunity. Plasmacytoid DCs (pDCs) originate from lymphoid precursors and produce type I interferons (IFNs) in response to pathogens. A20 is considered as a negative regulator of toll-like receptor (TLR) signaling pathways, in which *Toxoplasma gondii*- derived profilin (TgPRF) is a TLR11/12 ligand recognised by DCs to stimulate their maturation/activation. Little is known about contributions of A20 to changes in biological properties of pDCs. The present study, therefore, explored whether pDC functions are influenced by A20. To this end, bone marrow cells were isolated and cultured with Flt3L to attain CD8DCs, CD11bDCs and pDCs and followed by challenge with TgPRP in the presence or absence of *A20* siRNA. Expression of maturation markers were analysed by flow cytometry, and secretion of inflammatory cytokines by ELISA, cell migration by a transwell migration assay and expression of signalling molecules by western blotting. As a result, treatment with *A20* siRNA enhanced activations of IκB-α and STAT-1, leading to increases in expressions of maturation markers and cytokine productions as well as migration of TgPRP-treated pDCs, while mature CD11bDCs produced at higher levels of TNF-α and IL-6 only. In addition, functions of CD8DCs remained unaltered following A20 silencing. The effects of A20 on pDC maturation and activation were completely abolished by IKK inhibitor and partially blunted by fludarabine. In conclusion, the inhibitory effects of A20 on pDC functions are expected to affect the immune response in *T*. *gondii* infection.

## Introduction

Dendritic cells (DCs) are professional antigen-presenting cells (APCs) originated from a common bone marrow progenitor and involved in the induction of T cell-mediated adaptive immunity. The two major subsets of DCs including plasmacytoid DCs (pDCs) and conventional DCs (cDCs, subdivided into CD8DCs and CD11bDCs) express different toll-like receptors (TLRs), therefore play distinct roles in immunity regarding their interaction with pathogens [1]. The cDCs (CD11c^+^ cells) exhibit antigen recognition, cytokine production, and antigen presentation, at levels higher than pDCs and are potent inducers of effector T cells in response to infection, whereas pDCs tend to mediate immune tolerance rather than immunity [2]. pDCs originated from lymphoid precursors are CD11c^low^ B220^high^ expressing cells and the main producers of type I interferons (IFNs) in response to pathogens [3]. In addition, cDCs exhibit a high phagocytic capacity, whereas pDCs are only poorly phagocytic and absent in peripheral tissues under noninflammatory condition [4]. Recruitment of pDCs is frequently seen in viral infections and autoimmune disease such as systemic lupus erythematosus [5].

*Toxoplasma gondii*-derived profilin (TgPRF) is a toll-like receptor (TLR) 11/12 activating ligand of immune cells including DCs in mice and recognised by TLR5 in humans [6, 7]. TgPRF contributes to actin-dependent gliding motility and cellular invasion for *T*. *gondii* [8]. A binding of DCs with this ligand triggers a protective interleukin (IL)-12 response to induce T helper 1 cells (Th1) to secrete IFN-γ mediated through MyD88 and NF-κB activations [8–10], therefore, depletion of DCs renders mice susceptible to *T*. *gondii* infection [11]. Another studies reveal that mice lacking *TLR11* have dramatically reduced production of IL-12 [12] and *MyD88*- or *IFN-γ*-deficient mice exhibit dramatic susceptibility to *T*. *gondii* infection [13, 14]. A similar study on pDCs shows that infection with *T*. *gondii* up-regulates expressions of MHC class II and costimulatory molecules as well as cell migration to induce proliferation of naive CD4^+^ T cells [10] and these cells involve in controlling *T*. *gondii* infection in the initial stages [10].

A20 is considered as a negative regulator of nuclear factor (NF)-κB-dependent functions of many different cell types in response to lipopolysaccharide (LPS) [15] and inflammatory cytokines [16]. In mice, deficiency of A20 leads to hyperactivation of immune cells and autoimmune diseases [15]. *A20*-deficient mice display severe inflammation, cachexia and premature mortality [17]. Mice lacking A20 in DCs fail to LPS tolerance as they die within 6 hours after injection of LPS [15]. In humans, A20 also plays an important role in inhibiting inflammatory diseases and cancer [18, 19]. An inactivated expression of A20 is found frequently in leukemia [19].

Little is known about the role of A20 in the regulation of functions of DCs upon challenge with TgPRF. The present study has thus been performed to elucidate whether A20 participates in the regulation of biological functions of DCs. To this end, DCs were exposed to TgPRP and expression of maturation markers, cytokine release, and migration were determined. Activities of IκB-α and STAT-1 were assessed in TgPRP-stimulated DCs to determine the mechanism underlying the regulation of A20 in DCs.

## Materials and methods

### Mice

BALB/c mice were purchased from Taconic Farms (Hudson, NY, USA) and housed in a specific pathogen-free facility at Institute of Genome Research. The animals had free access to food and drinking water. Animal care and experimental procedures were performed according to the Vietnamese law for the welfare of animals and were approved by the ethical committee of Institute of Genome Research.

### Bone marrow-derived DCs

The methods used in this study were previously described elsewhere [20]. BALB/c mice were anesthetized with isoflurane gas and bone marrow cells were flushed out of the cavities from the femur and tibia with PBS. Cells were washed twice with RPMI-1640 and seeded out at a density of 4 x 10^6^ cells per 60-mm dish. Cells were cultured for 8 days in RPMI-1640 (GIBCO) containing: 10% FCS, 1% penicillin/streptomycin, 1% glutamine, 1% non-essential amino acids (NEAA) and 50μm β-mercaptoethanol. Cultures were supplemented with Flt3L (200 ng/mL, Peprotech) on days 3 and 6. CD8DCs, CD11bDCs and pDCs were isolated by using CD8^+^DC, CD11b^+^DC and plasmacytoid DC isolation kits, respectively (Militeny Biotech). For maturation, DC subsets were cultured with TgPRP (5μg/ml, Sigma Aldrich) in the presence or absence of fludarabine (80 μM, Sigma Aldrich) or IKK inhibitor (2 μM, Sigma Aldrich) for 20 hours.

### Transfection of DCs with siRNA

Control- or *A20*-targeted siRNA (pre-designed siRNA, Applied Biosystems) was transfected into DCs (2 x 10^5^ cells/1ml) with the help of Lipofectamine RNAiMAX Reagent (Invitrogen) according to the manufacturer’s recommendations. Cells were incubated for 48 h at 37°C, 5% CO_2_. After washing three times with PBS, cells were used for experiments.

### Immunostaining and flow cytometry

Cells (10^6^) were incubated in 100 μl FACS buffer (phosphate buffered saline (PBS) plus 0.1% FCS) containing fluorochrome-conjugated antibodies at a concentration of 10 μg/ml. A total of 5 x 10^4^ cells were analysed. The following antibodies (all from eBioscience) were used for staining: mouse IgG isotype control, anti-mouse CD11c, anti-mouse CD86, anti-mouse CD40 and anti-mouse I-A/I-E. After incubating with the Abs for 60 minutes at 4°C, cells were washed twice, resuspended in FACS buffer and analysed with flow cytometry (FACSAria Fusion, BD Biosciences).

### Cytokine quantification in cell supernatants

DC subsets were transfected with *A20* siRNA and followed by stimulating with TgPRP in the presence or absence of fludarabine and IKK inhibitor. Cell culture supernatant was collected and stored at -80°C until use for ELISA. For concentration analysis of IL-6, IL-10, IL-12p40, TNF-α and IFN-γ commercially available ELISA kits (eBioscience) were used according to the manufacturer’s instructions.

### Western blotting

CD11bDCs and pDCs (5 x 10^6^ cells each) were washed twice in PBS, and lysed in RIPA-1 buffer. Lysates were stored at -80°C until used for western blotting. The lysates were separated by 10% SDS-polyacrylamide gels, and blotted on polyvinylidene fluoride membrane. The blots were blocked with blocking buffer. Then the blots were probed overnight with anti-p-IκBα, anti-p-p38, anti-p-ERK1/2, anti-p-STAT-1 (727), anti-p-STAT-3 and anti-GAPDH (Santa Cruz) in blocking buffer, washed 5 times, probed with HRP anti-rabbit or anti-mouse secondary antibody (Amersham) for 1h at RT, and washed final 5 times. Antibody binding was detected with the enhanced ECL Plus kit (GE Healthcare).

### DC migration assay

DC subsets were washed twice with PBS and suspended in RPMI 1640 medium. Migration was assessed in triplicate in a multiwell chamber with a pore diameter size of 8 μm (BD Falcon). The cell suspension (50000 cells/ml) was placed in the upper chamber to migrate into the lower chamber in which either CCL19 (300ng/ml, Peprotech) or medium alone as a control for spontaneous migration were included. The chamber was placed in a 5% CO_2_, 37°C incubator for 4h. The cells that migrated into the lower chamber were collected and counted under a light microscope. The mean number of spontaneously migrated cells was subtracted from the total number of migrated cell and migration was considered by calculating the percentage of migrating cell related to input.

### Statistics

Data are provided as means ± SEM, *n* represents the number of independent experiments. All data were tested for significance using Student’s unpaired two-tailed *t*-test or ANOVA and only results with p < 0.05 were considered statistically significant.

## Results

### A20 inhibits pDC maturation

**Fig 1:**
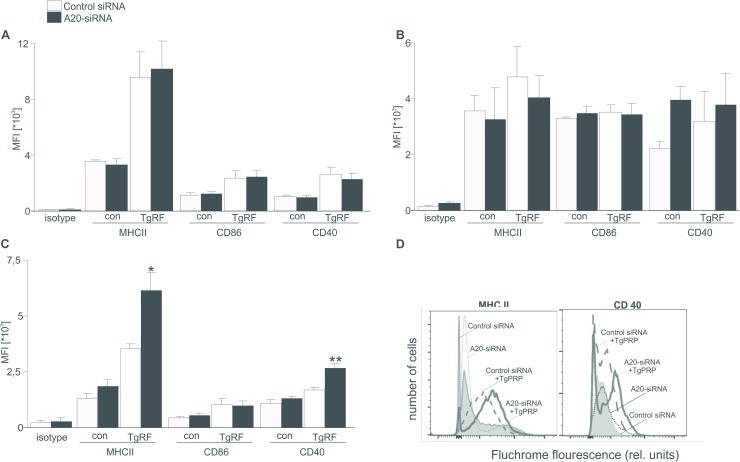
Effect of A20 on maturation of DCs. **A-C.** Arithmetic mean ±SEM (n = 5–7) of mean fluorescence intensity (MFI) of MHCII^+^, CD86^+^ and CD40^+^ expressing control or TgPRP (5μg/ml)-treated CD8DCs (A), CD11bDCs (B) and pDCs (C), which were either treated with control siRNA or *A20* siRNA. *(p<0.05) and ** (p<0.01) indicate significant differences from control siRNA-treated cells, (ANOVA). **D.** Representative FACS histograms depicting MHC II and CD40 fluorescences were attained from control or TgPRP (5μg/ml)-treated pDCs, which were either treated with control siRNA or *A20* siRNA.

To ask whether A20 influences in expressions of surface markers on CD8DCs, CD11bDCs and pDCs. Cells were transfected with control or *A20* siRNA and followed by TgPRP treatment. DC subsets were collected and stained for IgG isotype control, MHC class II, costimulatory molecule CD86 and CD40. Challenge with TgPRP increased percentages of MHCII^+^, CD86^+^ and CD54^+^ expressing CD8DCs and pDCs (Fig 1A and 1C), and did not affect expression of these markers on CD11bDCs (Fig 1B).

In the absence of A20, expressions of MHCII and CD40, but not CD86 on TgPRP-mature pDCs were significantly enhanced, whereas expressions of MHCII, CD86 and CD40 on both CD8DCs and CD11bDCs were unaltered (Fig 1A–1C), indicating that A20 prevented expressions of MHC II and CD40 markers on pDCs.

### A20 inhibits cytokine secretion in pDCs and CD11bDCs

**Fig 2:**
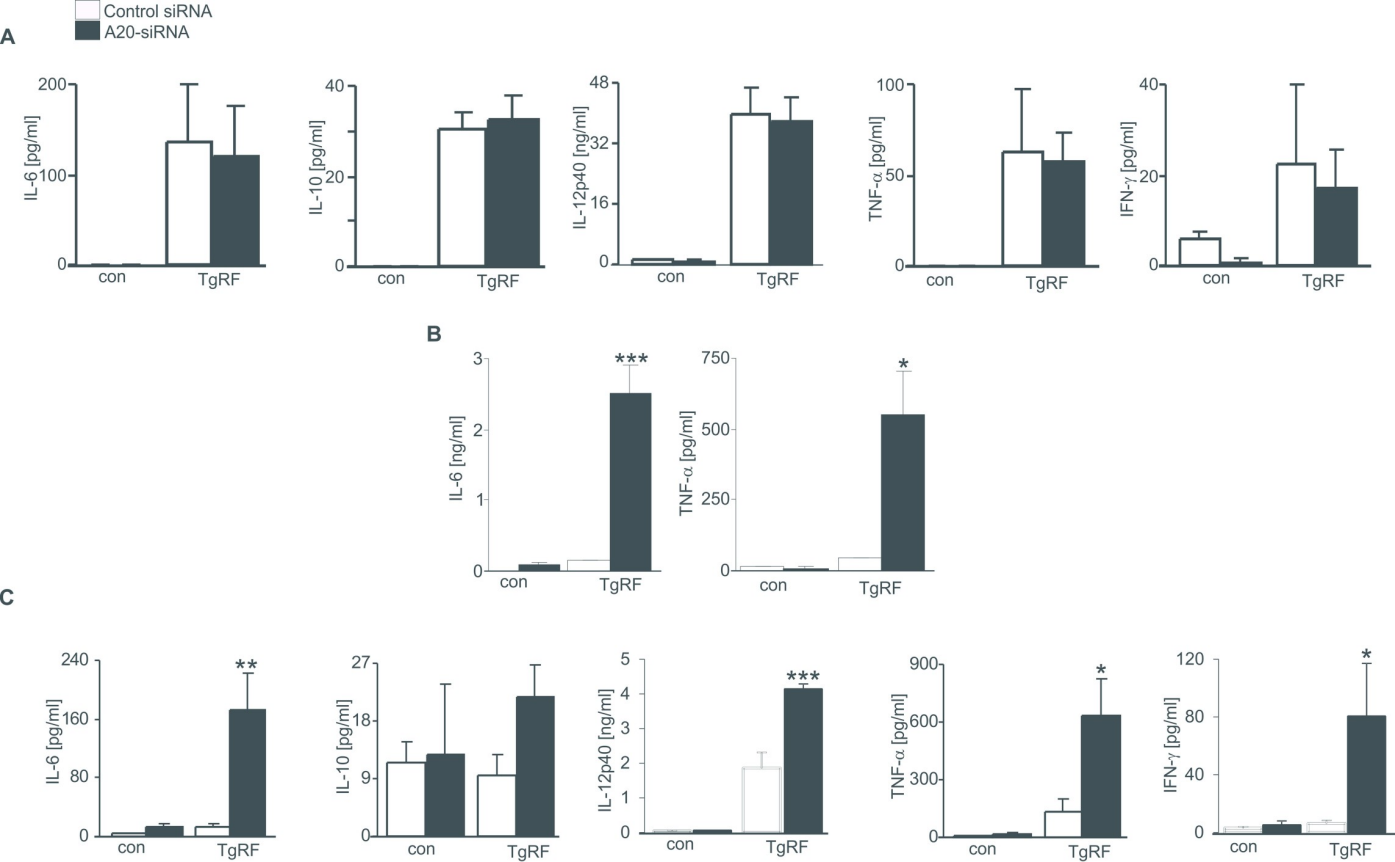
Effect of A20 on cytokine productions by DCs. **A-C.** Arithmetic means ± SEM (n = 5–7) of IL-6, IL-10, IL-12p40, TNF-α and IFN-γ secretions from control or TgPRP (5μg/ml)-treated CD8DCs (A), CD11bDCs (B) and pDCs (C), which were either treated with control siRNA or *A20* siRNA. IL-10, IL-12p40 and IFN-γ by CD11bDCs were measured and not detected, therefore only IL-6 and TNF-α are presented in the Fig 2B. *(p<0.05), ** (p<0.01) and *** (p<0.001) indicate significant differences from control siRNA-treated cells, (ANOVA).

We next examined cytokine productions secreted by CD8DCs, CD11bDCs and pDCs when exposed with TgPRP. As illustrated in Fig 2, challenge of control DC subsets with TgPRP increased cytokine productions IL-6, IL-10, IL-12p40, TNF-α and IFN-γ secreted by CD8DCs and pDCs (Fig 2A and 2C) and slightly enhanced levels of IL-6 and TNF-α in CD11b^+^ DCs. All the cytokines were measured but IL-10, IL-12p40 and IFN-γ could not be detected in CD11b^+^ DCs and therefore only IL-6 and TNF-α are presented in the Fig 2B. Consistently, recent studies report that cytokines IL-12p40 and IFN-γ are not produced by CD11b^+^DCs during *T*. *gondii* infection [21, 22].

To investigate the role of A20 in the regulation of cytokine secretion by DC subsets, we observed that treatment of the cells with *A20* siRNA resulted in the enhanced release of IL-6 and TNF-α by both pDCs and CD11bDCs and levels of IL-12p40 and IFN-γ by pDCs only (Fig 2A–2C). In addition, IL-10, IL-12p40 and IFN-γ by TgPRF-treated CD11bDCs were measured and not detected (with data not shown). The evidence indicated that A20 inhibited inflammatory reaction in pDCs and partially in CD11bDCs when exposed to TgPRP.

### A20 inhibits migration of pDCs

**Fig 3:**
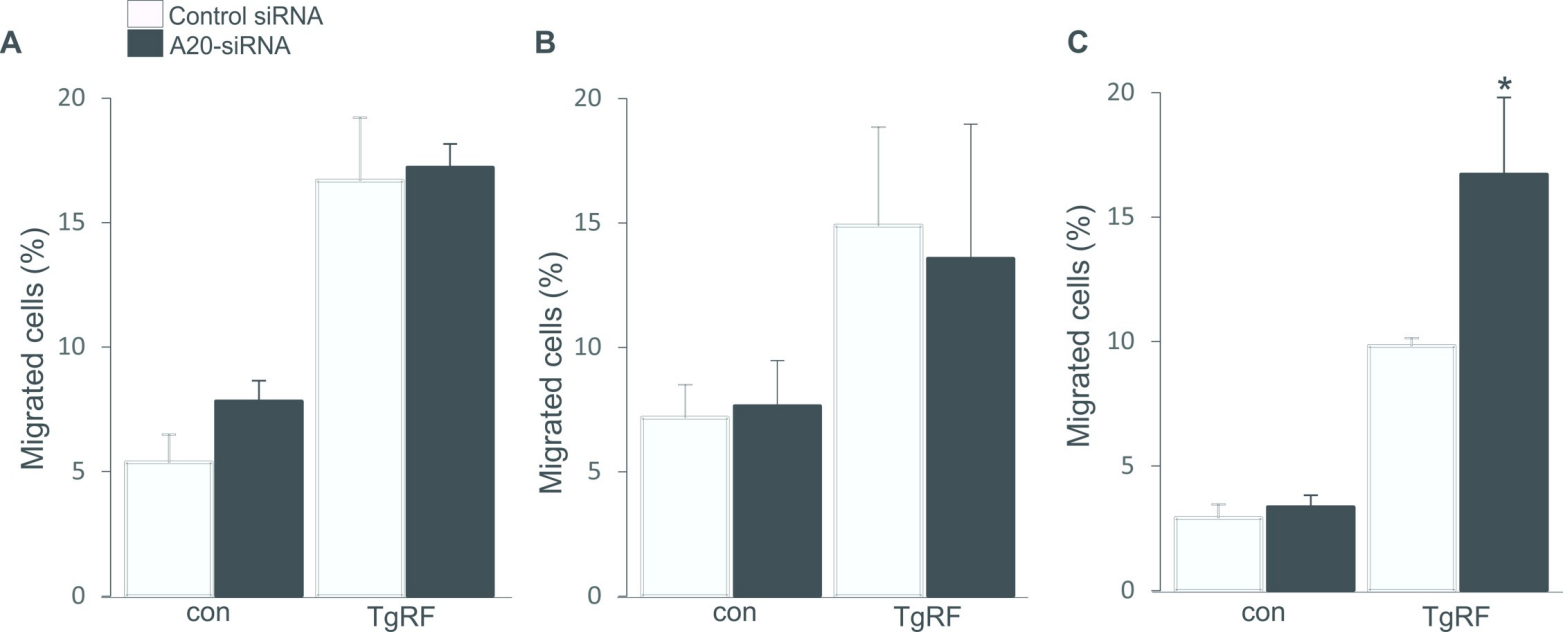
Effect of A20 on DC migration. **A-C.** Arithmetic means ± SEM (n = 4–5) of percentages of migrated control or TgPRP (5μg/ml)-treated CD8DCs (A), CD11bDCs (B) and pDCs (C), which were either treated with control siRNA or *A20* siRNA. *(p<0.05) indicates significant difference from control siRNA-treated cells, (ANOVA).

A recent study shows the role of A20 in supressing migration of cancer cells [23]. Similar to our results attained from expression of maturation markers, challenge of DC subsets with TgPRP also led to enhanced migration of CD8DCs and pDCs, but not CD11bDCs (Fig 3A–3C). In the absence of A20, migration of mature pDCs only was further enhanced (Fig 3C), indicating that migration of TgPRP-matured pDCs was inhibited by the presence of A20.

### A20 inhibits activations of IκB-α and STAT-1 signallings in pDCs

**Fig 4:**
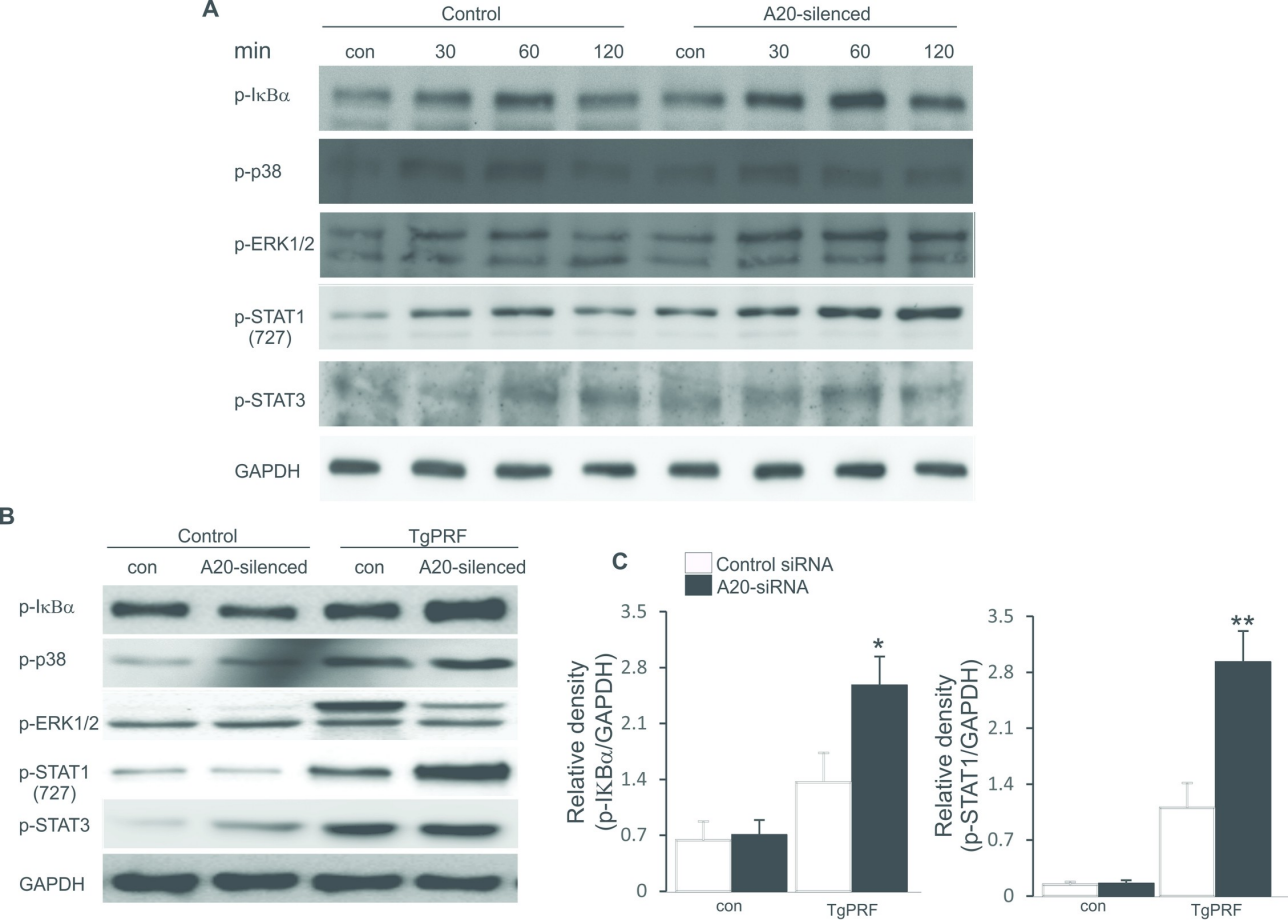
Effect of A20 on signalling pathways in CD11bDCs and pDCs. **A.** Original Western blot of CD11bDCs were either treated with TgPRP (5μg/ml) for indicated time points: 30, 60, 120 min or left untreated (control) in the absence or presence of *A20* siRNA. Protein extracts were analysed by direct Western blotting using antibodies directed against p-IκB-α, p-p38, p-ERK1/2, p-STAT-1 (727), p-STAT-3 and GAPDH. **B.** Original Western blot of pDCs were either treated with TgPRP (5μg/ml) for 60 min or left untreated (control) in the absence or presence of *A20* siRNA. Protein extracts were analysed by direct Western blotting using antibodies directed against p-IκB-α, p-p38, p-ERK1/2, p-STAT-1 (727), p-STAT-3 and GAPDH. **C.** Arithmetic mean ±SEM (n = 5) of the abundance of p-IκB-α and p-STAT1 proteins as the ratios of p-IκB-α/GAPDH and p-STAT1/GAPDH in control or TgPRP (5μg/ml)-treated pDCs, which were either treated with control siRNA or *A20* siRNA. *(p<0.05) and ** (p<0.01) indicate significant differences from control siRNA-treated cells, (ANOVA).

Since A20 supressed the maturation and activation of pDCs and partially inflammatory reaction in CD11bDCs, therefore we examined expressions of signalling molecules involved in the regulation functions of pDCs and CD11bDCs. CD11bDCs and pDCs were treated with TgPRP for one hour and total cell protein was extracted by using RIPA-1 lysis buffer. As shown in Fig 4B and 4C, treatment with *A20* siRNA resulted in the enhanced phosphorylation of IκB-α and STAT-1 in TgPRP-challenged pDCs, whereas activations of these molecules in *A20*-silenced CD11bDCs was slightly increased, but not reaching to the significance (Fig 4A). In this study, the IFN-γ level was not produced in the supernatant of both control and *A20*-silenced pDCs after one hour of TgPRF treatment (data not shown). Therefore, the phosphorylation of STAT-1 was not stimulated by IFN-γ produced by pDCs in the cell culture. In addition, activations of other signaling molecules including p38MAPK, pERK1/2 and pSTAT-3 remained unaltered when the cells were transfected with *A20* siRNA. The results showed that A20 contributed to an inhibitory effect on the activations of IκB-α and STAT-1 signallings in pDCs.

### A20 inhibits functions of pDCs through IκB-α and partial STAT-1 signallings

**Fig 5:**
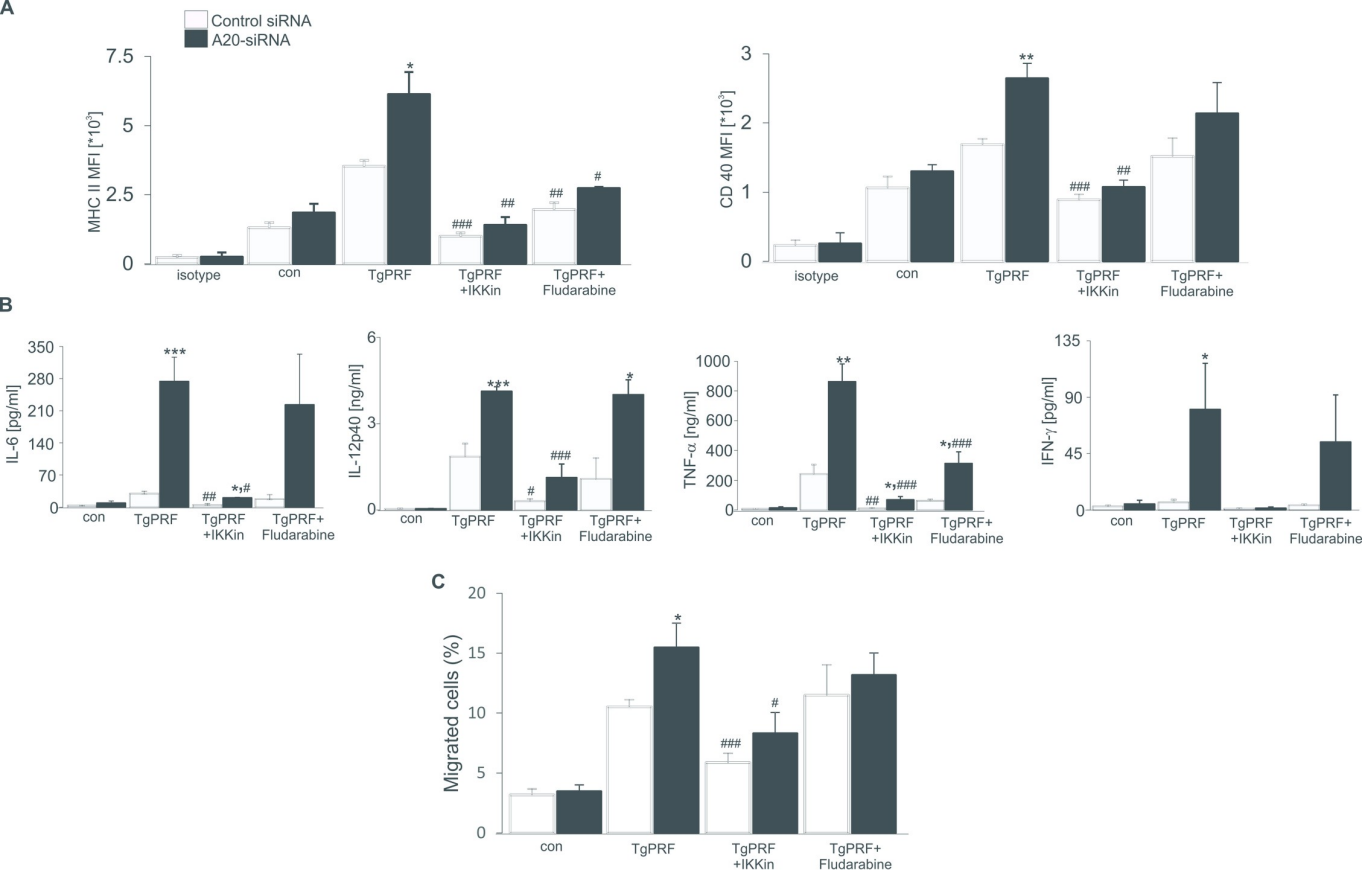
A20 inhibits functions of pDCs through IκB-α and partial STAT-1 signallings. **A.** Arithmetic mean ±SEM (n = 5–7) of mean fluorescence intensity (MFI) of MHCII^+^ (left) and CD40^+^ (right) expressing control or TgPRP (5μg/ml)-treated pDCs, which were either treated with control siRNA or *A20* siRNA in the presence or absence of IKK inhibitor (2 μM) or fludarabine (80 μM). *(p<0.05) and ** (p<0.01) indicate significant differences between control and *A20*-silenced pDCs. ^≠^ (p<0.05), ^≠≠^ (p<0.01) and ^≠≠≠^ (p<0.001) indicate significant differences from TgPRF-treated pDCs (ANOVA). **B.** Arithmetic mean ±SEM (n = 4–7) of IL-6, IL-12p40, TNF-α and IFN-γ secretions from control or TgPRP (5μg/ml)-treated pDCs, which were either treated with control siRNA or *A20* siRNA in the presence or absence of IKK inhibitor (2 μM) or fludarabine (80 μM). *(p<0.05), ** (p<0.01) and *** (p<0.001) indicate significant differences between control and *A20*-silenced pDCs. ^≠^ (p<0.05), ^≠≠^ (p<0.01) and ^≠≠≠^ (p<0.001) indicate significant differences from TgPRF-treated pDCs (ANOVA). **C.** Arithmetic mean ±SEM (n = 4–7) of percentages of migrated control or TgPRP (5μg/ml)-treated pDCs, which were either treated with control siRNA or *A20* siRNA in the presence or absence of IKK inhibitor (2 μM) or fludarabine (80 μM). *(p<0.05) indicates significant difference between control and *A20*-silenced pDCs. ^≠^ (p<0.05) and ^≠≠≠^ (p<0.001) indicate significant differences from TgPRF-treated pDCs (ANOVA).

We next performed experiments to ask whether changes in biological properies of pDCs is mediated through IκB-α and STAT-1 activations. The pharmacological inhibitors of STAT-1 signaling fludarabine and IκB-α signaling IKK inhibitor were added in pDC culture, expressions of maturation markers, cytokine productions and cell migration were examined. As shown in Fig 5A, the inhibitory role of A20 on expressions of MHCII and CD40 on pDCs was significantly blunted in the presence of fludarabine or IKK inhibitor, although fludarabine significantly supressed expressions of MHCII on control and *A20*-silenced pDCs only.

Besides, the contents of the pro-inflammatory cytokines IL-6, IL-12p40, TNF-α and IFN-γ were checked when mature pDCs were treated with fludarabine or IKK inhibitor. As shown in Fig 5B, the role of IKK inhibitor in inhibiting cytokine productions of IL-6, IL-12p40, TNF-α and IFN-γ were observed, while fludarabine significantly reduced level of TNF-α only in both control and *A20*-silenced mature pDCs. Importantly, the supressing effect of A20 on IFN-γ secretion only by pDCs was abolished, whereas the differences in releases of IL-6, IL-12p40 and TNF-α between control and *A20*-silenced mature pDCs remained unaltered (Fig 5B).

Finally, the increased migration of *A20*-silenced pDCs was also abolished when the cell culture was added IKK inhibitor or fludarabine (Fig 5C). Based on the results attained, we revealed that A20 prevented maturation and activation of pDCs through NF-κB and partial STAT-1 signalling pathways.

## Discussion

To our knowledge, we showed for the first time that downregulation of A20 expression resulted in increases in pDC maturation/activation and inflammatory reaction in CD11bDCs in response to TgPRF. Similar to previous reports [8, 10, 24, 25], we also illustrate that expressions of the maturation markers are increased in TgPRP-stimulated CD8DCs and pDCs and unaltered in TgPRP-stimulated CD11bDCs. Upon challenge with TgPRP, CD8^+^DCs initiate the immune reaction, while CD11bDCs lack the IRF8 transcription factor, which induces the releases of IL-12p40, IL-10 and IFN-γ [24– 26]. Importantly, we found that in the absence of A20, expressions of MHCII^+^ and CD40^+^, but not CD86^+^ molecules on TgPRP-matured pDCs were significantly enhanced, whereas percentages of MHCII^+^, CD86^+^ and CD40^+^ expressing CD8DCs and CD11bDCs remained unaltered (Fig 1). Besides, the release of IL-6, IL-12p40, TNF-α and IFN-γ cytokines in *A20*-silenced mature pDCs was higher compared to control pDCs (Fig 2). This finding is supported by other reports indicating that IL-12 and IFN-γ are considered the major mediators of host resistance to *T*. *gondii* infection [8, 10, 22] and other cytokines such as TNF-α secreted by DCs contribute to latter host protection from this infection [27]. In addition, the phenotypic and functional maturation of DCs are affected by Ca2^+^ influx, an important determinant for cell migration [28], which was increased in pDCs only when the cells were transfected with *A20* siRNA (Fig 3). The role of A20 in inhibiting cell migration has been reported by other authors [16, 29]. The evidence illustrated that A20 participated in inhibiting maturation and activation of pDCs and might be involved in the development of *T*. *gondii* infection.

Interestingly, TgPRP-stimulated CD11bDCs displayed similar expressions of the maturation markers and cytokine productions as control cells, however cytokines IL-6 and TNF-α secreted by these cells were significantly enhanced following A20 silencing (Fig 2B). Our results are in line with the studies on DCs, which indicates that CD11b^+^DCs do not produce cytokines IL-12p40 and IFN-γ during *T*. *gondii* infection [21, 22, 24, 25]. Differently, our recent study showed that A20 suppresses the release of IL-12 and TNF-α, but not IL-6 by CD11bDCs in the response with LPS [15], whose binding to TLR4 to trigger transcription of multiple genes associated in the regulation of maturation/differentiation and activation. Therefore, A20 in CD11bDCs modulated cytokine productions of IL-6 and TNF-α, rather than the antigen-presenting and stimulatory capacities and might partially involve in the immune regulation of cytokine against *T*. *gondii* infection.

More importantly, the phosphorylations of IκB-α and STAT-1, but not STAT-3 were significantly elevated in *A20-*silenced pDCs and slightly increased in *A20*-silenced CD11bDCs in the exposure with TgPRP (Fig 4), therefore IKK inhibitor and fludarabine were added to pDC culture to suppress the activation of IκB-α and STAT-1 signalling molecules. As expected, the IKK inhibitor and fludarabine completely reversed the differences in expression of maturation markers, migration capacity and IFN-γ production only between control and *A20*-silenced pDCs (Fig 5). In many studies, A20 has been shown to be a negative regulator of NF-κB-mediated cell functions [15, 16, 30], although the role of A20 in the regulation of *T*. *gondii* infection is unknown. Recently in several cell types, A20 is also known to have an inhibitory effect on STAT-1 activation [16, 18, 23], which prevents the death from *T*. *gondii* infection in mice [22].

Upon LPS treatment, A20 participates in suppressing CD11bDC functions through NF-κB and ERK signalling pathways [15]. Differently, we observed that A20 downregulated the phosphorylation of NF-κB and STAT-1 in pDCs, but did not affect these signalling pathways in CD11bDCs in the exposure to TgPRP. Therefore, CD11bDCs and pDCs may exploit different mechanisms of pathogen recognition to induce immune reaction. Clearly, CD11bDCs are characterized by high expression of CD11c and have high capacity to induce T cell-mediated immunity to secrete type I IFNs [15, 31], whereas pDCs are CD11c^low^ expressing cells that can induce tolerogenic immune responses [2] and directly produce type I IFNs in response to various stimuli [1, 10].

In conclusion, results obtained from the present study revealed that the negative regulation of TgPRP-induced pDC maturation and activation by A20 was dependent on NF-κB and partially STAT-1 signallings. The effects of A20 on pDCs are expected to affect the immune response in *T*. *gondii* infection.
